# Recurrence of Acne inversa of the penis – two-stage reconstruction with MatriDerm^®^ and split-thickness skin graft 

**DOI:** 10.3205/iprs000193

**Published:** 2025-12-22

**Authors:** Antek Nicklas, Vlad Stefan, Adrian Dragu

**Affiliations:** 1 University Center for Orthopedics, Trauma and Plastic Surgery, Department of Plastic and Hand Surgery, University Hospital Carl Gustav Carus at the TU Dresden, Dresden, Germany

**Keywords:** Acne inversa, penis reconstruction, STSG, CEM, MatriDerm

## Abstract

**Background:**

The authors report a case of a 42-year-old male patient who presented with a relapse of penile acne inversa five years after a split-thickness skin graft without use of collagen-elastin-matrix (CEM). After unsatisfactory pharmacological treatment, resection of the affected skin, negative pressure wound therapy (NPWT), CEM application, and split-thickness skin graft (STSG) were performed in several steps.

**Methods:**

We performed initial debridement and consecutive NPWT. After achieving a clean wound bed, a CEM (1 mm MatriDerm^®^; MedSkin Solutions Dr. Suwelack AG, Germany) was applied, followed by split-thickness skin grafting.

**Results:**

During a 19-month follow-up, the patient presented with a very good functional and cosmetic outcome. Under intravenous antibiotic therapy and intensive wound care, we achieved complete recurrence-free status in the genital area at the 19-month follow-up. The subjective quality of life almost tripled from 3.2 to 9.3 on the numeric analog scale (NAS).

**Conclusion:**

Complex reconstruction procedures of penile defects in acne inversa can be avoided if there is a well-perfused wound bed. Coverage of the defect with MatriDerm^®^ and split-thickness skin grafting may lead to long-term aesthetically satisfactory results with high patient satisfaction. We have monitored one patient over 19 months, who presented with stable penile soft tissue coverage and no signs of relapse. We anticipate that coverage of penile defects using MatriDerm^®^ and split-thickness skin grafts may prevent or at least delay the need for complex reconstruction in cases of recurrence.

## Zusammenfassung

**Hintergrund:**

Die Autoren berichten über einen Fall eines 42-jährigen männlichen Patienten, der fünf Jahre nach einer Spalthauttransplantation ohne Verwendung einer Kollagen-Elastin-Matrix (CEM) ein Rezidiv einer penilen Acne Inversa hatte. Nach unzureichender pharmakologischer Behandlung wurden eine Resektion der betroffenen Haut, Vakuumtherapie (NPWT), die Anwendung von CEM und eine Spalthauttransplantation (STSG) in mehreren Schritten durchgeführt.

**Methoden:**

Es wurde zunächst ein radikales Debridement durchgeführt, gefolgt von einer Unterdrucktherapie. Nach Erreichung eines sauberen Wundbettes wurde eine CEM (1 mm MatriDerm^®^; MedSkin Solutions Dr. Suwelack AG, Deutschland) angewendet, gefolgt von einer Spalthauttransplantation.

**Ergebnisse:**

Nach einem 19-monatigen Follow-up zeigte der Patient ein sehr gutes funktionelles und kosmetisches Ergebnis. Unter intravenöser Antibiotikatherapie und intensiver Wundpflege wurde der vollständige, rezidivfreie Zustand im Genitalbereich beim 19-monatigen Follow-up erreicht. Die subjektive Lebensqualität verdoppelte sich fast von 3,2 auf 9,3 auf der Numerischen Analogskala (NAS).

**Schlussfolgerung:**

Komplexe Rekonstruktionsverfahren bei penilen Defekten infolge von Acne inversa können vermieden werden, wenn ein gut durchblutetes Wundbett vorhanden ist. Die Defektdeckung mit MatriDerm^®^ und Spalthauttransplantation kann zu langfristig ästhetisch zufriedenstellenden Ergebnissen mit hoher Patientenzufriedenheit führen. Wir haben einen Patienten über 19 Monate beobachtet, bei dem eine stabile Weichteildeckung des Penis ohne Anzeichen eines Rezidivs vorlag. Wir gehen davon aus, dass die Deckung peniler Defekte mit MatriDerm^®^ und Spalthauttransplantaten ein Rezidiv mit der Notwendigkeit einer komplexen Rekonstruktion verhindern oder zumindest zeitlich verzögern kann.

## Introduction

Acne inversa (also known as Hidradenitis suppurativa) is a rare, chronic debilitating inflammatory skin disease. According to the Dessauer definition, it is a deeply localized, painful inflammatory skin lesion that occurs in areas rich in apocrine glands, particularly in the axillary and inguinal or anogenital regions [1], [2]. In older studies, the prevalence of acne inversa was reported to be 0.4 and 4% [3]. In a representative study of the French population, the prevalence was reported to be 1% [4]. Penile manifestations with the extreme variant of elephantiasis are much rarer and are considered a rarity. The male-to-female ratio for acne inversa ranged from 1:2.7 to 1:3.3 [4], [5], [6]. In contrast, men are more commonly affected in the perineal region [7]. The average age at first manifestation is 23 years [3].

The pathogenesis of the disease is multifactorial and not fully understood. Some authors attribute bacterial superinfections of ingrown hair follicles to be the cause [8]. While others consider blockages of apocrine sweat glands to be responsible for the subsequent granulomatous inflammation [7]. Risk factors include smoking and obesity, which are associated with a more severe course [6]. The Hurley staging system was developed as a way to classify the severity of Hidradenitis Suppurativa (HS) symptoms. It consists of three stages that help to track the progression of HS and guide treatment decisions: Stage 1: Solitary or multiple isolated abscess formation without scarring or sinus tracts. Stage 2: Recurrent abscesses, single or multiple widely separated lesions, with sinus tract formation. Stage 3: Diffuse or broad involvement, with multiple interconnected sinus tracts and abscesses [9].

Surgical intervention remains the best treatment option for severe cases from Stage II onwards. In Stage I and preoperatively, topical therapies and laser treatments are possible and recommended options [10], [11]. 

The defect coverage of complex wounds generally follows the reconstructive ladder, which has evolved into the reconstructive elevator [12], [13]. Adhering to these principles, starting with the simplest procedure, is essential when planning defect coverage. Although complex wounds, especially in patients with acne inversa, can be treated with complex, resource-intensive, and expensive flaps such as fascio-myocutaneous Dartos flaps. Simpler therapy options with adjunctive provide good alternatives that can be performed easier and do not require microsurgical expertise. The reason for using a CEM, such as MatriDerm^®^ (MedSkin Solutions Dr. Suwelack AG, Germany) lies in its ability to support the growth of keratinocytes and fibroblasts, mitigate scar contractures in stress zones [14], [15], [16].

The use of MatriDerm^®^ for the reconstruction of various soft tissue defects is well documented in the literature [17], [18], [19]. Regarding long-term outcomes, earlier studies have shown that compared to other dermal substitute materials such as Integra^®^ (Integra Lifescience Corporation, Plainsboro, New Jersey, USA), MatriDerm^®^ is more frequently associated with scar contractures but less often with foreign body reactions [9].

In the present case, it concerns a recurrence of acne inversa in the sensitive urogenital area – an indication for which there is currently little data on the use of MatriDerm^®^. An exception is the study by Ludolph et al., which documents the successful use of MatriDerm^®^ in acne inversa-associated and Fournier’s gangrene-associated defects in the urogenital region, achieving good functional and aesthetic results [20].

Furthermore, collagen-elastin matrices (CEM) have already been used more frequently in urogenital reconstructive surgery in general, especially in complex urethral strictures, where the matrix serves as dermal substitute tissue to support coverage after plastic expansion [21]. These positive experiences from related indications underscore the potential of MatriDerm^®^ in reconstructive procedures following extensive excisions due to acne inversa recurrence.

We present the case of a 42-year-old male patient with a recurrence of penile acne inversa, five years after radical excision and split-thickness skin grafting without the application of a CEM matrix. After exhausting conservative therapy and in the context of the recurrence, the patient reported increasing psychological limitations in his daily life. We performed another radical debridement, NPWT at –75 mmHg (3M™), and the application of 1 mm monolayer MatriDerm^®^ followed by STSG.

## Material and methods

**Figure 1:**
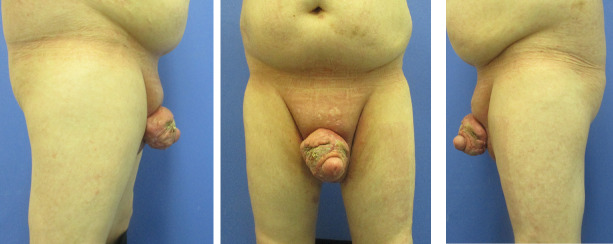
Preoperative situation of the recurrence

The patient presented with a recurrence of acne inversa in the penile area, five years after the initial surgery (Figure 1 (Fig. 1)). The disease had been present in the patient for seven years with progressive findings. Other affected areas include both axillae. The condition is classified as stage III in the Hurley classification. The diagnosis had been histologically confirmed five years earlier. Topical treatments by the involved dermatologists did not lead to any improvement.

**Figure 2:**
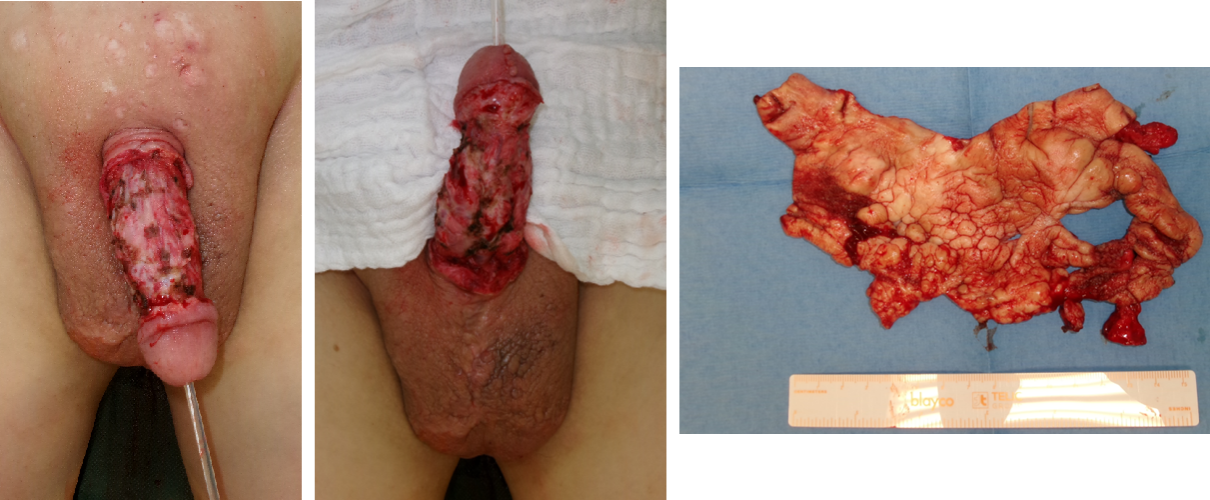
Intraoperative finding: 1^st^ debridement

Initially, we performed radical debridement of all altered skin areas up to the inner foreskin and started NPWT (Figure 2 (Fig. 2)). After five days, another debridement was performed along with full-thickness skin grafting and additive NPWT. After removing the vacuum dressing, the full-thickness skin graft showed partial necrosis.

**Figure 3:**
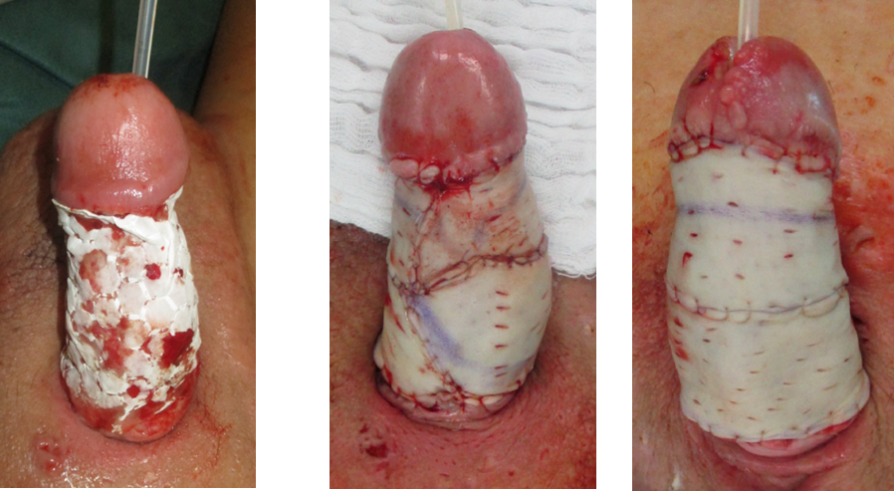
Intraoperative application of MatriDerm^®^ and skin graft

**Figure 4:**
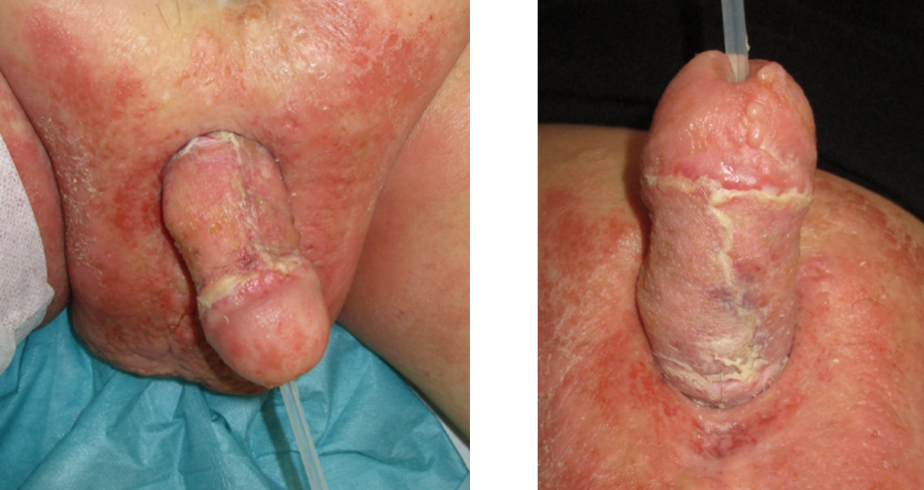
Five days postoperative

We performed another tangential debridement and the application of 1-mm monolayer MatriDerm^®^ without suturing. Subsequently, we restarted NPWT for another seven days. With a well-granulated wound bed and fully integrated MartiDerm^®^, the STSG (0.3 mm thickness; MESH ratio 1:1, harvested from left thigh) was performed, followed by another vacuum therapy (Figure 3 (Fig. 3)). After removing the vacuum dressing after five days, the split-thickness skin was 95% adherent and healed (Figure 4 (Fig. 4)).

**Figure 5:**
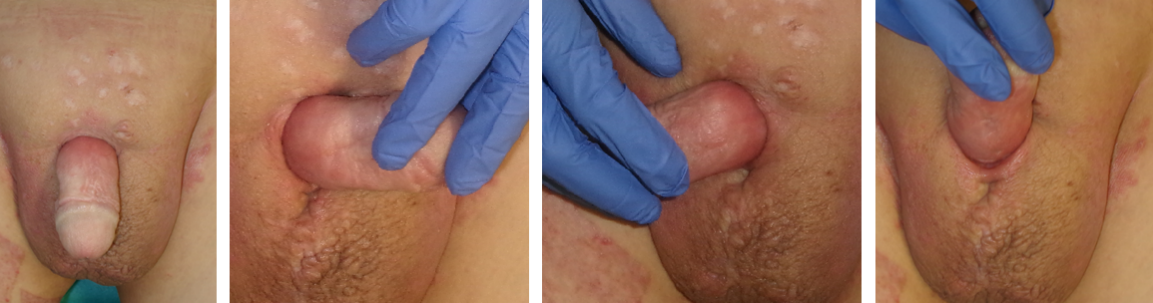
Results 6 months postoperative

**Figure 6:**
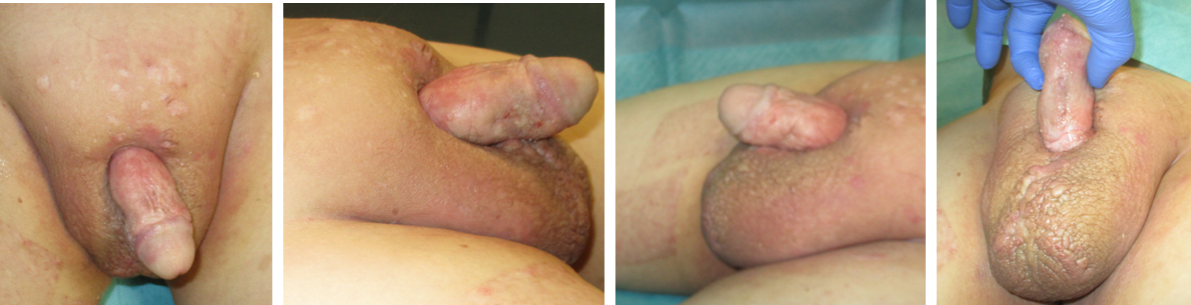
Results 12 months postoperative

The total hospital stay was 38 days, during which five operations under general anesthesia were performed. Check-ups were conducted at 2 weeks, 4 weeks, 3 months, 6 months (Figure 5 (Fig. 5)), and 12 months (Figure 6 (Fig. 6)).

At the last follow-up after 12 months, there was no indication of a recurrence in the penile area. The patient’s preoperative and postoperative quality of life was assessed using the Numeric Analog Scale (NAS).

## Results

The average duration of the five performed operations was 64 minutes. During the hospital stay, we administered antibiotic therapy with Cefuroxime 1.5 g intravenously three times a day for 14 days, followed by Ciprofloxacin 750 mg per os twice a day for another four days. These antibiotics were used targeted to the pathogen upon detection of* Staphylococcus aureus*. The same spectrum of pathogens had already been identified five years earlier.

In the histopathological examination the diagnosis of acne inversa (Hurley III), made five years earlier, was again confirmed. No recurrence in the penile area occurred 19 months postoperatively. Conservative therapy in the axillary areas on both sides also resulted in a subjective improvement.

At the follow-up examination, the patient showed a very good functional and cosmetic outcome, allowing even the resumption of a normal sex life. His subjective quality of life value on the NAS almost tripled from 3.2 to 9.3.

## Discussion

Plastic surgical reconstructions in patients with stage III acne inversa according to Hurley present a significant challenge for the surgeon. The use of MatriDerm^®^ in combination with a staged split-thickness skin grafting represents a simple reconstruction option with a decreased risk of recurrence. Reconstructions in the penile area for acne inversa patients using MatriDerm^®^ and split-thickness skin grafts have not been previously described. Particularly due to the need for dermal elasticity during erection, the use of collagen-elastin matrices is a good choice to ensure erection without an extra tension. Previous studies from our clinic also suggest that the staged application of MatriDerm^®^ in such areas appears to be effective. The significant improvement in the patient’s quality of life described in our case demonstrates that the use of MatriDerm^®^ followed by split-thickness skin grafting leads to a good outcome in this rare disease.

In case of secondary failure of simple reconstruction using CEM and split-thickness skin grafts, larger reconstructions, such as fascio-myocutaneous Dartos flaps, which have significantly higher donor site morbidity, can still be used later. However, the prerequisite for a successful reconstruction with CEM and split-thickness skin graft remains the radical debridement with a complete excision of the affected skin areas and subcutaneous tissue including glandular tissue [22], [23]. Additionally, the application of NPWT before and after CEM application is sensible and established to ensure the integration of the CEM [16]. In addition, the guideline-compliant antibiotic therapy conducted in our case is also essential for therapeutic success. 

After completed wound healing, dermatological follow-up treatment should be continued in our experience.

CEM such as MatriDerm^®^ or Integra^®^ are often associated with high costs and are therefore not ubiquitously available in all healthcare systems worldwide. This limited availability restricts their widespread use, particularly in resource-limited regions, and thus represents a significant barrier to broad clinical application.

In summary, the application of MatriDerm^®^ after radical excision of the affected areas with staged split-thickness skin grafting represents an effective but expensive method for defect reconstruction in penile acne inversa recurrences with high patient satisfaction.

## Limitation

This case study has several limitations that should be considered when interpreting the results. Firstly, the investigation is based on a single patient case, which limits the generalizability of the findings. To reliably assess the efficacy and safety of using MatriDerm^®^ for acne inversa defects in the urogenital area, further studies with larger patient cohorts are necessary.

Furthermore, the quality of the quality-of-life measurement in this study is limited, as primarily a simple scale was used, which is only partially suitable for capturing quality of life. In addition, alternative dermal substitute materials and their possible applications in the urogenital region were not examined in detail in this work. A comparative analysis of these products could provide further insights within the scope of a study and support clinical decision-making. To assess the sustainability and long-term outcome, annual follow-up appointments are conducted at our university outpatient clinic. In additional follow-ups after 19 months, no recurrence or deterioration of findings was observed.

Finally, a longer follow-up period would be advisable to make statements about the durability of the reconstruction as well as the risk of recurrence.

Overall, these limitations highlight the need for further research to better define the role of MatriDerm^®^ in urogenital reconstruction for acne inversa and to optimize treatment strategies and associated therapeutic algorithms.

## Notes

### Institutional review board statement

The study was conducted in accordance with the Declaration of Helsinki and approved by the Ethics Committee of TU Dresden.

### Informed consent

Written informed consent to publish the paper and the image files has been obtained from the patient.

### Competing interests

The authors declare that they have no competing interests.
